# The Effect of Surface-Modified Gold Nanorods on the Early Stage of Embryonic Development and Angiogenesis: Insight into the Molecular Pathways

**DOI:** 10.3390/ijms222011036

**Published:** 2021-10-13

**Authors:** Nouf N. Mahmoud, Zain Zaki Zakaria, Hadeel Kheraldine, Ishita Gupta, Semir Vranic, Maha Al-Asmakh, Ala-Eddin Al Moustafa

**Affiliations:** 1 Faculty of Pharmacy, Al-Zaytoonah University of Jordan, Amman 11733, Jordan; 2 College of Medicine, QU Health, Qatar University, Doha 2713, Qatar; semir.vranic@gmail.com; 3 Biomedical Research Center, Qatar University, Doha 2713, Qatar; zain.zakaria@qu.edu.qa (Z.Z.Z.); hk1805332@student.qu.edu.qa (H.K.); maha.alasmakh@qu.edu.qa (M.A.-A.); 4 Biomedical and Pharmaceutical Research Unit, QU Health, Qatar University, Doha 2713, Qatar; ishugupta28@gmail.com; 5 Department of Biomedical Sciences, College of Health Sciences, QU Health, Qatar University, Doha 2713, Qatar

**Keywords:** gold nanorods, chick chorioallantoic membrane, angiogenesis, embryogenesis, apoptosis

## Abstract

Gold nanorods have been implicated in several biomedical applications. Herein, the effect of two surface-modified gold nanorods on the early stages of embryogenesis and angiogenesis was investigated using avian embryos at three days and their chorioallantoic membrane (CAM) at five days of incubation. We found that gold nanorods (GNR) modified with PEGylated phospholipid moiety show a high mortality rate in embryos after four days of exposure compared to GNR modified with PEGylated cholesterol moiety. Meanwhile, our data revealed that surface modified-GNR significantly inhibit the formation of new blood vessels in the treated CAM model after 48 h of exposure. Moreover, we report that surface-modified GNR significantly deregulate the expression of several genes implicated in cell proliferation, invasion, apoptosis, cellular energy metabolism, and angiogenesis. On the other hand, our data point out that GNR treatments can modulate the expression patterns of JNK1/2/3, NF-KB/p38, and MAPK, which could be the main molecular pathways of the nanorods in our experimental models.

## 1. Introduction

Embryogenesis, a regulated embryonic development, shares remarkable cellular and molecular similarities with cancer, particularly in cellular proliferation, cell differentiation/dedifferentiation, cell migration/invasion, and angiogenesis [1,2]. Multiple key signaling pathways involved in embryonic development are often dysregulated in cancer, promoting tumor progression [2]. Similarly, dysregulated angiogenesis is correlated with several diseases caused or exacerbated by pathological angiogenesis [3]; for example, tumor angiogenesis plays a crucial role in cancer progression where cell invasion, metastasis, and excessive cancer cell growth are initiated or accelerated [4]. Multiple pro-angiogenic and anti-angiogenic factors contribute to the formation of new blood vessels [5,6,7]; thus, targeting angiogenesis to prevent cancer progression provides appreciable therapeutic benefits. Various anti-angiogenesis drugs have been approved for cancer therapy by targeting different pro-angiogenetic regulatory factors and other pathways [8]; however, challenges including drug resistance and severe adverse effects may limit their clinical applications [9].

Today, modulating angiogenesis via nanotechnology has been the focus of increased interest due to the unique advantages provided by nanoparticles, such as their high surface area and selective targeting into tumors, resulting in long half-life, enhanced efficacy, and reduced adverse effect [10]. Among nanomaterials, gold nanoparticles (GNP) demonstrated promising anti-cancer effects and other biomedical applications due to their unique physical and plasmonic properties [11,12]. GNP has been utilized as angiogenesis modulators in multiple studies, and the activation or inhibition of angiogenesis by GNP is strongly correlated with their surface functionalization and formulation. For example, a previous study demonstrated the ability of GNP to modulate (inhibit or enhance) angiogenesis in the chorioallantoic membrane (CAM) model by conjugating different types of peptides to the nanoparticles [13]. The angiogenesis modulation activity of GNP was demonstrated in several *in vivo* and *ex-vivo* models; for example, GNP showed an anti-angiogenesis effect in a mouse model inoculated with human colorectal cancer [14], in an animal model of melanoma [15], and a mouse model of ovarian tumor [16].

Many angiogenesis studies of GNP have been conducted using CAM as an *in vivo* model. CAM, a rich vascular tissue of the avian embryo, is a simple, low-cost, and excellent *in vivo* model for exploring the angiogenesis modulation effect of novel therapeutic drugs and candidates [13,17]. It has been revealed that diverse molecular mechanisms and pathways contribute to GNP’s anti-angiogenic effects, the VEGF-A/VEGFR pathway being the main molecular target [18]. Furthermore, Vimalraj et al. found that biogenic GNP demonstrates anti-cancer activity and exhibits a significant anti-angiogenetic role in the CAM model by downregulating Ang-1/Tie 2 pathway [19].

Most GNP angiogenesis studies were conducted using spherical GNP, while rod-shaped GNP has been primarily utilized in bioimaging, therapeutic and diagnostic applications [20]. Previous reports indicate that biological responses towards GNP, including cytotoxicity, cellular internalization, and bio-distribution, are strongly correlated with GNP’s shape, size, and surface chemistry [21,22]. Our recent work displayed that the interaction of gold nanorods (GNR) with human skin or cancerous cells is considerably correlated with the nanoparticles’ surface modification; GNR conjugated with polyethylene glycol (PEG)-phospholipid moiety exhibited enhanced uptake into human skin tissue [23] and breast cancer cell lines, promoting several apoptotic pathways [24,25], and modulating the production of metabolites responsible for cellular energy metabolism [26]. On the other hand, GNR conjugated with a cholesterol moiety was successfully utilized as a nanocarrier to deliver anti-cancer and antifungal agents [27,28]. This study addresses the possible anti-angiogenic activity of two surface-modified GNR to support their previously observed anti-cancer outcome. In addition, this study provides insight into the potential toxicity of these two GNR preparations during the early stages of embryo development and the proposed molecular mechanisms and pathways underlying their activity.

## 2. Results

### 2.1. Synthesis, Functionalization and Characterization of GNR

**Figure 1:**
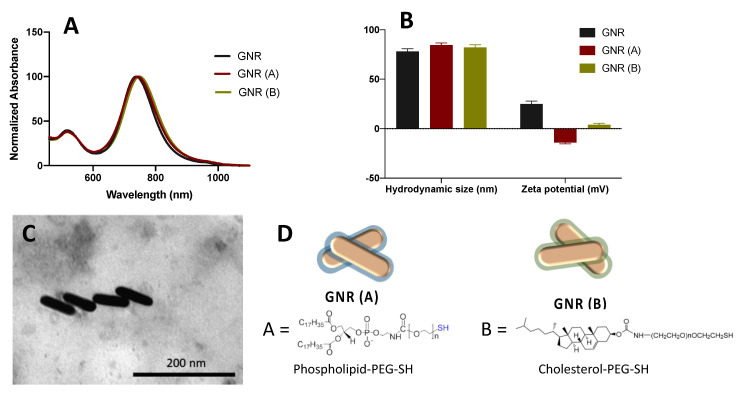
(**A**) UV-Vis absorption spectra of GNR before and after surface functionalization with PEG-phospholipid GNR (A) and PEG-Cholesterol GNR (B). (**B**) Hydrodynamic sizes and effective surface charges of GNR before and after surface functionalization with PEG-phospholipid; GNR (A) and PEG-Cholesterol; GNR (B). (**C**) TEM image of GNR (A) confirms the nanorods’ size and shape. (**D**) Surface coating materials of GNR (A) and GNR (B).

GNR was successfully synthesized and stabilized using a mixture of surfactants (CTAB and sodium oleate). The prepared nanorods demonstrated typical longitudinal and transverse peaks at ~523 nm and 760 nm, respectively, indicating their excellent colloidal stability (Figure 1A). The surface functionalization of the nanorods was performed to displace CTAB moiety and consequently reduce its toxicity and increase the colloidal stability of the nanorods. Surface modification of the nanorods with phospholipid GNR (A) and cholesterol moieties GNR (B) resulted in stable nanorods suspensions with slightly shifted longitudinal peaks (Figure 1A). The average hydrodynamic size of GNR before surface functionalization was ~78 nm, with a surface charge of +25 mV due to the adsorption of positively charged CTAB molecules onto the surface of nanorods (Figure 1B). The average hydrodynamic sizes of GNR (A) and GNR (B) are ~84 nm and 82 nm, respectively, and their effective surface charges are −12 mV and +4 mV, correspondingly, which confirmed the successful surface functionalization of GNR with DSPE-PEG-SH or Cholesterol-PEG-SH moieties (Figure 1B). GNR imaging by TEM was performed for GNR (A) to confirm their rod shape with an average length and width of ~67 nm and ~18 nm, respectively. FTIR spectroscopy and ^1^H NMR analysis confirmed the surface conjugation with phospholipid and cholesterol moieties as described previously [23,27]. Surface coating materials for GNR (A) and GNR (B) are presented in Figure 1D.

### 2.2. The Effects of GNR (A) and GNR (B) on the Early Stage of Embryonic Development

**Figure 2:**
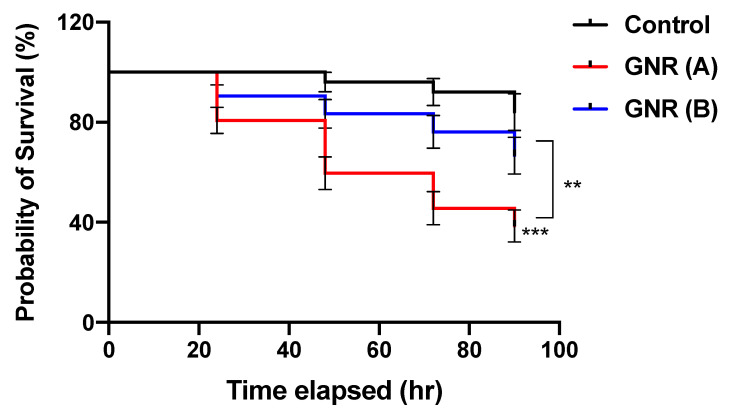
Kaplan Meier survival curves of embryos treated with GNR (A) and GNR (B) compared to the control. GNR (A)-exposed embryos exhibit significantly lower survival events than GNR (B)-treated embryos and control. ** *p* < 0.01, *** *p* < 0.001.

To explore the impact of GNR (A) and GNR (B) on the early stage of embryonic development, the mortality rate and survival probability of the exposed embryos were estimated using the chicken embryo model, as described in the Materials and Methods section. The results presented in Table 1 indicate that chicken embryos treated with GNR (A) exhibited a higher mortality rate after four days of incubation (61.4%) than those treated with GNR (B) (33.5%) or untreated embryos (16%). Furthermore, the Kaplan Meier survival curve in Figure 2 indicates that embryos exposed to GNR (A) exhibited significantly lower survival probability over the treatment course compared to GNR (B) (*p* < 0.01) and control (*p* < 0.001). Furthermore, there is no significant difference in the survival probability of GNR (B) compared to the control.

### 2.3. Angiogenesis Effect of GNR (A) and GNR (B) Using the CAM of the Chicken Embryo Model

**Figure 3:**
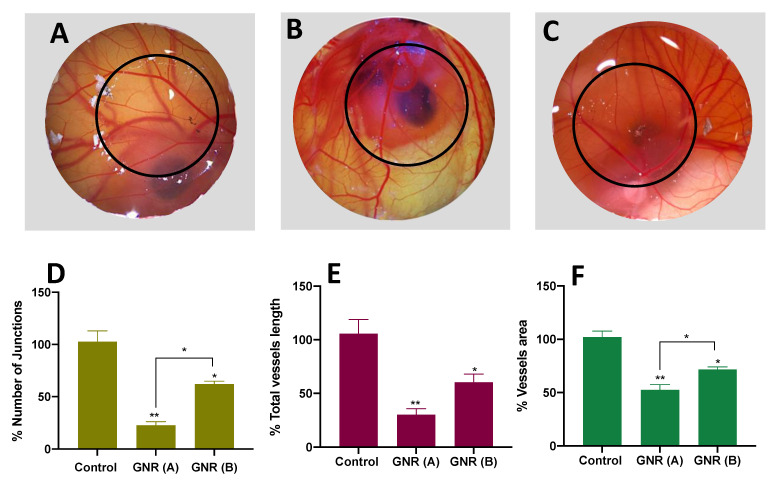
Angiogenesis of the CAM of chicken embryos: (**A**) untreated; (**B**) treated with GNR (**A**), and (**C**) treated with GNR (**B**). GNR (**A**) and (**B**) significantly inhibit the formation of new blood vessels compared to untreated CAM. Percentage of the number of junctions (**D**); total vessels length (**E**) and vessels area (**F**) in exposed areas compared to unexposed ones in the control CAM, and those treated with GNR (**A**) or GNR (**B**). Both GNR treatments significantly reduced the number of junctions, total vessels length, and vessels area in the exposed CAM compared to the control. Data are represented as mean ± SD, * *p* < 0.05, ** *p* < 0.01. Magnification: 10×.

The impact of surface modified-GNR on the formation of new blood vessels was explored using the CAM model at 5 days of incubation. Interestingly, the results demonstrate that GNR decorated with phospholipid GNR (A) or cholesterol GNR (B) moieties significantly inhibited the formation of blood vessels in the CAM model after 48 h of exposure compared to the control. More specifically, GNR (A) had a more pronounced anti-angiogenesis effect compared to GNR (B) (Figure 3A–C). These data were confirmed by quantifying the number of junctions, total blood vessel length, and blood vessels area in the exposed zone (labeled with black circles in Figure 3A–C) compared to the unexposed zone. Both GNR (A) and (B) treatments showed a significant percentage reduction in the number of junctions compared to the control (*p* < 0.01 and *p* < 0.05, respectively) (Figure 3D). Interestingly, GNR (A) exhibited a significant percentage reduction in the number of junctions compared to GNR (B) (~77% vs. ~38%, *p* < 0.05; Figure 3D). Moreover, both GNR (A) and (B) treatments revealed a significant percentage reduction in total vessels length (~70%, *p* < 0.01, and ~40%, *p* < 0.05), respectively; Figure 3E), and in vessels area (~50%, *p* < 0.01, and ~30%, *p* < 0.05, respectively; Figure 3F) compared to the control. In addition, GNR (A) displayed a significant percentage reduction in the vessels area compared to GNR (B) (~50% vs. ~30%, *p* < 0.05; Figure 3F).

### 2.4. Effects of GNR (A) and GNR (B) on Gene Expression in Different Tissues from Exposed Chicken Embryos

**Figure 4:**
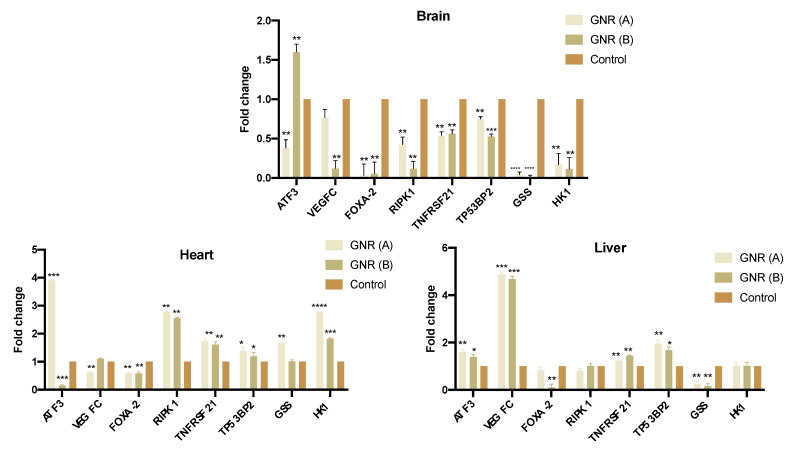
RT-PCR analysis of genes implicated in apoptosis, angiogenesis, and cellular energy metabolism using the brain, heart, and liver tissues of chicken embryos treated with GNR (A) or (B) compared to the control. Data are represented as mean ± standard deviation (SD), * *p* < 0.05, ** *p* < 0.01, *** *p* < 0.001, **** *p* < 0.0001.

The expression patterns of a set of genes implicated in cell proliferation, apoptosis, angiogenesis, and cellular energy metabolism were explored. The results in Figure 4 revealed that the expression level of *ATF3* was significantly upregulated in the brain (*p* < 0.01) and heart tissues (*p* < 0.001) upon treatment with GNR (B) and (A), respectively. Furthermore, GNR (A) and (B) significantly upregulated the expression level of *ATF3* in the liver tissue of the exposed embryos (*p* < 0.001 and *p* < 0.05; respectively). Regarding genes involved in angiogenesis, migration, and invasion, the expression of *VEGF-C*, was significantly downregulated in the brain tissue upon treatment with GNR (B) (*p* < 0.01), and in the heart tissue when treated with GNR (A) (*p* < 0.01); however, *VEGF-C* was overexpressed in the liver for both treatments. Furthermore, the expression of *FOXA-2* was significantly decreased in all organs upon treatment with GNR (*p* < 0.01). Both GNR treatments significantly upregulated the expression of *RIPK1* in the heart tissue of treated-embryos (*p* < 0.01); however, its expression was not significantly modulated in the liver. Moreover, the expression of *TNFRSF21* and *TP53BP2*, which are implicated in apoptosis signaling pathways [29,30], were significantly upregulated in the heart (*p* < 0.01) and liver (*p* < 0.01) tissues of exposed embryos with GNR (A) or (B). On the other hand, the expression of *GSS*, which is implicated in the cellular energy metabolism [31], was significantly reduced in the brain (*p* < 0.0001) and liver (*p* < 0.01) tissues of chicken embryos treated with GNR (A) or (B). Similarly, the expression of *HK1*, a key regulator factor of cellular glycolysis [32], was significantly reduced in the brain tissue (*p* < 0.01) of embryos treated with GNR (A) or (B); however, its expression was upregulated in the heart and was not significantly modulated in the liver of the exposed embryos.

### 2.5. Effects of GNR (A) and GNR (B) on the Morphology of Cultured Chicken Embryo Fibroblasts

**Figure 5:**
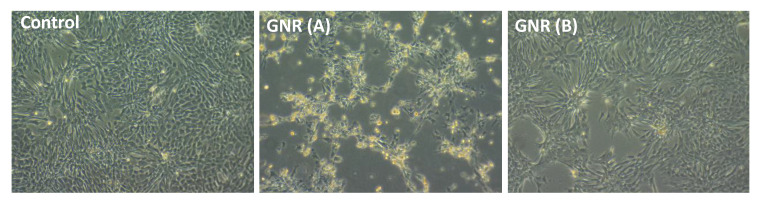
Effect of GNR (A) and (B) treatments on the microscopic morphology of cultured chicken embryo fibroblasts after 48 h of exposure compared to untreated cells (control). GNR (A) induced significant morphological alterations in the embryonic cells compared to GNR (B) and untreated cells.

As shown, untreated cells produced a confluent layer and showed typical morphological characteristics of spindle-shaped fibroblasts (Figure 5). However, a high percentage of the cells became round upon treatment with GNR (A) for 48 h, and many of them lost their membrane integrity and developed condensed cytoplasm with possible apoptotic bodies (Figure 5). Treatment with GNR (B) resulted in slight morphological alterations of cells compared to untreated control cells (Figure 5).

### 2.6. Effects of GNR (A) and GNR (B) on Protein Expression Patterns Analysis of JNK 1/2/3, NF-KB p65, and P38 MAPK in Embryonic Fibroblasts

**Figure 6:**
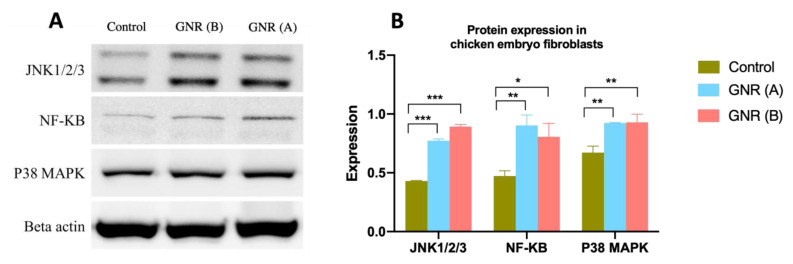
Western blot (**A**) and quantification of bands (**B**) of the expression of JNK 1/2/3, NF-KB p65, and P38 MAPK in cultured chicken embryonic fibroblasts upon treatment with GNR (A) and (B). The results revealed a significant increase in the expression of these key regulatory factors of apoptosis and autophagy signaling pathways. Data are represented as mean ± SD, * *p* < 0.05, ** *p* < 0.01, *** *p* < 0.001.

To further determine the molecular pathways of GNR treatments in our experimental models, the expression patterns of JNK 1/2/3, NF-KB p65, and P38 MAPK were analyzed in embryonic fibroblasts upon treatment with GNR (A) or GNR (B). Our data revealed that both treatments significantly increased the expression of JNK1/2/3 (*p* < 0.001), NF-KB (*p* < 0.05), and P38 MAPK (*p* < 0.01) in treated cells compared to their matched control (Figure 6).

## 3. Discussion

GNP and particularly non-spherical types such as GNR are frequently utilized in biomedical applications due to their unique features related to their particle size, surface chemistry, and plasmonic properties [33]. GNP’s surface modification modulates their biological responses such as cytotoxicity, cellular uptake, bio-distribution, and cellular death modalities [34].

In this study, GNR of aspect ratio ~4 was synthesized using the seed-mediated method, and then successfully functionalized with a thiolated-PEGylated moiety of either phospholipid or cholesterol linker. The presence of PEG in both ligands has a crucial role in enhancing the colloidal stability of the functionalized nanorods; in addition, thiol has accelerated the surface ligand exchange process due to the high affinity of gold towards thiolated ligands [35]. This surface functionalization of nanorods enhances their colloidal stability and significantly reduces the concentration of CTAB, a toxic surfactant involved in the process of GNR synthesis [36].

We demonstrated in our previous work the cytotoxicity of GNR decorated with a phospholipid moiety (GNR (A)) towards a panel of breast cancer cell lines and their modulation effect on several regulatory factors involved in cellular apoptosis and energy metabolism [24,25,26]. Furthermore, GNR-decorated with a cholesterol moiety (GNR (B)) were utilized as an efficient nanocarrier for several drugs with relatively low cytotoxicity towards normal healthy cells [27,28,37]. In this study, we used the chicken embryo and its CAM to provide insight into the impact of these two GNR preparations on the early stage of embryogenesis and their possible anti-angiogenic activity to support their previously observed anti-cancer outcome, as normal development shares several major events with carcinogenesis. Additionally, this study can provide information about the possible toxicity of GNR during pregnancy, especially at the early stages since they could be used as drug delivery in pregnant patients.

Regarding the toxic impact of GNR exposure on the early developmental stages of exposed chicken embryos, our data point out that GNR (A) and (B) treatments represent ~3.8-fold and ~2.0-fold increase in the mortality rate, respectively, compared to unexposed embryos. Moreover, GNR (A) revealed a ~1.8-fold increase in the mortality rate compared to GNR (B), with a low survival probability.

Moreover, our results indicate that both GNR treatments have a significant anti-angiogenesis activity on the CAM model. Interestingly, GNR decorated with a phospholipid moiety, GNR (A), significantly retarded the formation of new blood vessels compared to GNR modified with a cholesterol ligand, GNR (B), particularly in terms of vessel junctions and vessels area. Such difference might be related to the nanorods’ cellular uptake; we recently reported that coating the nanorods with a phospholipid ligand has dramatically enhanced their cellular internalization into breast cancer cells [25] and Doxorubicin-resistant breast cancer cells (unpublished work). Although the uptake of GNR into the normal fibroblasts was not significant in our previous study, these results could not be conclusive on the *in-vivo* studies. The modulation effect of GNP on the normal physiological angiogenesis process was extensively investigated in the literature and was strongly correlated with surface modifications of the nanoparticles [18,38,39]. However, studying the implication of rod-shaped GNP on the angiogenesis process is rare in general. Pathological angiogenesis has a crucial role in the growth and progression of cancer cells, thus, suppressing such a process is considered one of the essential strategies in cancer therapy [40]. The anti-angiogenic activity of GNP is strongly correlated with their proposed anti-cancer properties. An earlier report found that GNP have an intrinsic anti-angiogenesis property via inhibiting heparin-binding growth factors and this effect was linked to their observed anti-tumor activity [16]. The anti-angiogenic effect of GNR in this study strongly supports their proposed anti-cancer activity towards breast cancer cell lines reported in previously published studies [24,25]. It is worth mentioning that although GNR (A) showed a significant anti-angiogenesis effect and high mortality rate toward embryos, their anti-cancer activity against breast cancer cells was observed even at concentrations much lower than that utilized in the current angiogenesis and embryogenesis studies [24,25].

To determine the gene targets of GNR treatments on embryogenesis and angiogenesis, the expression patterns of a set of genes implicated in cell proliferation, invasion, apoptosis, cellular energy metabolism, and angiogenesis in the exposed embryos were investigated. We found that the expression of *ATF3* was upregulated in some organs of the embryos upon treatment with GNR; *ATF3* regulates several cellular functions and is implicated in the cell cycle arrest and apoptosis. Several studies found that *ATF3* is downregulated in many human cancers [41,42]. Chen et al. found that *ATF3* acts as a tumor suppressor in hepatocellular carcinoma [43], and it has been reported that overexpression of *ATF3* is associated with the inhibition of cell proliferation and invasion [44].

On the other hand, our findings indicate that *VEGF-C* expression was decreased or not changed in the brain and heart upon treatment with GNR; *VEGF-C* plays a central role in vascular endothelial proliferation and migration and promotes angiogenesis and endothelial cell growth [45], and previous studies found that downregulation of *VEGF-C* inhibits tumor growth and metastasis by multiple mechanisms [46]. However, the expression of *VEGF-C* was increased in the liver tissue for both treatments, this could be explained by the previous observed novel hematopoietic function of *VEGF-C* in fetal erythropoiesis [47,48]. Moreover, our results point out that the expression of *FOXA-2*, was significantly downregulated in the organs of the exposed embryos. *FOXA-2* is associated with cell proliferation, invasion, and metastasis of various tumors [49], and a previous study found that *FOXA-2* is overexpressed in triple-negative/basal-like breast cancer cells and is associated with high relapse [50]. Other molecular pathways are responsible for the anti-angiogenesis activity of GNP, mainly modulating the VEGF-A/VEGFR pathway, pro-angiogenic factors, and inflammatory factors [18].

Furthermore, our data indicate that the expression of *RIPK1* was upregulated in the heart of the exposed chicken embryos upon treatment with GNR, while its expression was not significantly modulated in the liver. *RIPK1* plays a role in inflammation and necroptosis [51], and its role in cancer is complicated. Although necroptosis is considered as “fail-safe” mechanism that could prevent tumor development when apoptosis is compromised, the key factors of necroptosis could promote oncogenesis and cancer metastasis [51]. On the other hand, the expression of two apoptotic promotors, *TNFRSF21* [29], and *TP53BP2* [30], was significantly upregulated in the heart and liver of chicken embryos exposed to GNR treatments. These genes interact with other regulatory factors such as the p53 family and regulate the apoptotic pathways [52]. These results are in line with our previous study, which demonstrated that GNR (A) significantly upregulated the expression of *TP53BP2* in the MCF-7 breast cancer cell line and modulated the expression of other regulatory genes involved in several apoptotic pathways [25].

The observed downregulation of *ATF3*, *RIPK1*, *TNFRSF21,* and *TP53BP2* in some organs of exposed chicken embryos suggests a possible anti-apoptotic prevention effect of GNR treatments in the exposed embryos.

On the other hand, our data indicate that GNR treatments suppressed the expression of metabolic markers such as *GSS* in the brain and liver of treated chicken embryos. *GSS* is implicated in the synthesis of glutathione and its expression is elevated in various types of cancers such as breast and colon [31,53]. Similarly, *HK1*, a key regulatory metabolic marker for glycolysis and tumor metastasis [32], was downregulated in the brain tissue of exposed chicken embryos; however, its expression was upregulated in the heart of the exposed embryos, indicating a possible cardioprotective effect of the treatments [54]. These results agree with our previous findings; we found that GNR (A) significantly modulated several metabolites associated with cellular energy metabolisms causing dysfunction of the TCA cycle and glycolytic activity in the treated MCF-7 breast cancer cells [25].

To further explore the underlying molecular pathways of GNR treatments, we explored the expression patterns of JNK 1/2/3, NF-KB p65, and P38 MAPK in chicken embryonic fibroblast cells upon exposure to GNR. It’s worth noting that fibroblasts treated with GNR (A) demonstrated dramatic microscopic morphological changes compared to those treated with GNR (B). Our analysis reveals that both GNR treatments in our study significantly upregulated the expression of JNK 1/2/3, NF-KB p65, and P38 MAPK compared to control. The MAPK family is divided into three main groups: JNK, P38 MAPK, and extracellular-regulated kinase 1/2 (ERK1/2) [55]. JNK substrates and P38 MAPK are strongly correlated with cell growth and apoptosis and their activation induces cell apoptosis in cancer cells in several studies [56,57]. These results concur with our current data of *TP53BP2* in exposed chicken embryo tissues; it has been shown that the expression of *TP53BP2* activates the JNK and NF-KB pathways leading to apoptosis [29], as all three JNKs (JNK 1/2/3) stimulate apoptotic pathways [58].

On the other hand, the cellular response of the transcription factor NF-KB is convoluted. Although expression of NF-KB is generally associated with anti-apoptotic events, stimulation of NF-KB pathways could promote apoptosis and enhance the sensitivity towards apoptotic regulatory factors under certain conditions and in specific cell lines [59,60]. NF-KB is a heterodimer comprising p65 and p50 subunits [61]. It has been shown that activation of NF-KB plays a key role in apoptosis in chicken fibroblast cells; mycoplasmal nuclease induced apoptosis in chicken embryonic fibroblasts via activation of NF-KB pathway [62]; furthermore, mycoplasmal lipoproteins induced apoptosis in lymphocytes via activation of NF-KB [63]. In particular, it was found that NF-KB p65 overexpression in epithelial cells of human skin is associated with cell-cycle arrest [64]. However, we could not exclude the anti-apoptotic protection effect of the activated NF-KB pathway in treated chicken embryonic fibroblasts.

The overall results indicate that GNR treatments exert a significant anti-angiogenesis effect and promote several apoptotic pathways in the chicken embryo model and therefore induce toxicity at the early stage of embryogenesis, which shares several important biological events with carcinogenesis.

## 4. Materials and Methods

### 4.1. Synthesis, Functionalization, and Characterization of GNR

GNR was synthesized using the seed-mediated method as described previously with slight modifications [37,65]. Cetyltrimethylammonium bromide (CTAB, Sigma Aldrich, St. Louis, MO, USA) and sodium oleate (Sigma Aldrich, USA) were used as surfactants in the synthesis of the GNR. The obtained nanorods were functionalized with a PEGylated phospholipid moiety; DSPE-PEG-SH (Nanosoft polymers, Winston-Salem, NC, USA) by incubating twice-centrifuged GNR with the polymer (1 mg/1 mL of diluted GNR). The solution was mixed overnight, then centrifuged at 10,000 rpm for 8 min and the obtained pellets were suspended into ultrapure water [25]. Additionally, GNR were conjugated with a cholesterol moiety; Cholesterol-PEG-SH (Nanosoft Polymers, Winston-Salem, NC, USA) (2 mg/1 mL diluted GNR), and the solution was mixed overnight, then centrifuged at 10,000 rpm for 8 min (Hettich EBA 21 Centrifuge, Sigma Aldrich, St. Louis, MO, USA), and the obtained pellets were suspended into ultrapure water [27].

The phospholipid-modified GNR (GNR (A) and cholesterol-modified GNR (GNR (B) were characterized by UV-Vis absorption over the range of 400–1100 nm (spectrophotometer, UV-1800, Shimadzu, Japan), zeta potential, and hydrodynamic size (Nicomp Nano Z3000 particle size-zeta potential analyzer, USA) to confirm surface functionalization, and transmission electron microscope (Morgani 268 TEM, FEI, Amsterdam, The Netherlands) to confirm the nanoparticle’s shape. The surface coating of GNR was also confirmed by Fourier-transform infrared (FTIR) spectroscopy and Proton nuclear magnetic resonance (^1^H NMR) as described in our previous published work [23,25,27].

### 4.2. Evaluating the Effects of GNR (A) and GNR (B) Treatments on the Early Stage of Embryonic Development

Fertilized chicken embryos were bought from the Arab Qatari Company for Poultry Production and incubated at 37 °C with 60% humidity in a MultiQuip egg incubator. All procedures were ethically approved by the Institutional Bio-safety committee of Qatar University. Briefly, a small circular incision was made on the top of the eggshell and the membrane was carefully removed by adding ~200 μL of phosphate buffer saline (PBS) (Sigma-Aldrich, St. Louis, MO, USA) [66].

Embryos were divided into three groups and treated on day three of incubation as follows; the first group (*n* = 56) received ~12 µg of GNR (A), the second group (*n* = 42) received ~12 µg of GNR (B), and the third group (*n* = 25) received normal saline and served as control. The two types of GNR were placed on a glass coverslip exposed directly to the CAM and the eggs were sealed gently and incubated at 37 °C with 60% humidity for four days after treatment. The mortality rate was recorded daily, then, survived embryos were sacrificed on day four post-treatment, and their brains, hearts, and liver tissues were autopsied for RNA extraction and RT-PCR analysis.

### 4.3. Angiogenesis Assay Using the CAM Model

The chorioallantoic membrane (CAM) of the chicken embryos were treated with GNR (A) or GNR (B) on day five of incubation to evaluate the effect of the nanorods on the vascular development of exposed CAM. The synthesized GNR (~12 µg) was placed as described above. The vascular development of the CAM was examined after 24 and 48 h of exposure using a stereomicroscope and images were captured. Quantification of the total number of junctions, length of blood vessels, and vessels area of exposed CAM was performed using the AngioTool Software 0.6a and compared to unexposed areas [67,68].

### 4.4. Gene Expression by Real Time-PCR (qRT-PCR) Analysis

#### RNA Extraction

RNA extraction was performed from brain, heart, and liver tissues of nanoparticle-exposed embryos using the NucleoSpin TriPrep, Mini kit for RNA, DNA, and protein purification (Macherey-Nagel, Duren, Germany) as per the manufacturer’s protocol. Briefly, the sample was homogenized and lysed in Buffer RP1 (350 µL) and ß-mercaptoethanol (3.5 µL). The lysate was then filtrated using NucleoSpin Filter and 70% ethanol (350 µL) was added to the homogenized lysate. The lysate was transferred to a NucleoSpin TriPrep Column and centrifuged at 11,000 rpm for 30 s. The NucleoSpin TriPrep Column was transferred to a new collection tube and washed using Buffer RA2 once (200 µL) and Buffer RA3 twice (600 µL and 250 µL, respectively). The membrane of the NucleoSpin TriPrep Column was dried at room temperature and pure RNA was eluted using 60 µL of RNase-free water. RNA concentrations were obtained using the nanodrop reader (ThermoFisher Scientific, Waltham, MA, USA) and the samples were stored at −80 °C for further analysis.

### 4.5. qRT-PCR

The cDNA synthesis was performed using High-Capacity cDNA Reverse Transcription SuperscriptTM IV VILOTM Master Mix kit (ThermoFisher Scientific, Waltham, MA, USA), as per the manufacturer’s instructions. Following the cDNA synthesis, the RT q-PCR was performed using SYBR™ Green PCR Master Mix (Applied Biosystems, Waltham, MA, USA), and specific primers (Applied Biosystems, Waltham, MA, USA) were designed against the following genes of interest: Activating transcription factor 3 (*ATF3*), Vascular endothelial growth factor C (*VEGFC*), Forkhead box protein A2 (FOXA-2), Receptor-interacting serine/threonine-protein kinase 1 (*RIPK1*), TNF Receptor Superfamily Member 21 (*TNFRSF21*), Tumor Protein P53 Binding Protein 2 (*TP53BP2*), Glutathione Synthetase (*GSS*) and Hexokinase-1 (*HK1*), Table 2. The mRNA expression signal was read using RT-qPCR (QuantStudioTM 6 Flex RT-qPCR System), and the relative quantity calculation was performed using the 2−ΔCT method as described by Rao et al. [69], with the fold change being calculated regarding the expression of the housekeeping gene GAPDH.

### 4.6. Microscopic Evaluation for Morphological Changes of Embryonic Fibroblast Cells (EFCs) upon Exposure to GNR Treatments

EFCs were generated in our lab as described previously [66]. Cells were suspended in RPMI-1640 media (Thermo Fisher Scientific, Waltham, MA, USA) and supplemented with 10% fetal bovine serum (FBS; Invitrogen, Life Technologies, Waltham, MA, USA) and 1% PenStrep antibiotic (Thermo Fisher Scientific, Waltham, MA, USA), then incubated at 37 °C in a 5% CO_2_ atmosphere.

Cultured EFCs (~1 × 10^6^) were seeded and treated with GNR (A) and GNR (B) (~5.5 µg/mL, based on the viability study) and supplemented with 10% fetal bovine serum (FBS) for 48 h. Treated cells were visualized and their morphology was examined under the microscope after 48 h of incubation (Leica DMi1 inverted microscope, Leica Microsystems, Mannheim, Germany), then images of the cells were captured and compared to untreated ones.

### 4.7. Western Blot Analysis

This analysis was performed to investigate changes in protein expression of regulatory factors implicated in cellular functions related to cell apoptosis and metastasis. Briefly, cultured chicken embryo fibroblasts (~1 × 10^6^ cells) were seeded and treated with GNR (A) and GNR (B) (~5.5 µg/mL) and supplemented with 10% fetal bovine serum (FBS) for 48 h to enhance the colloidal stability of the nanorods [70]. The cell lysate was then collected, and an equal number of proteins were resolved in 10% SDS PAGE gel, then transferred onto PVDF membranes. Empty binding sites of the membranes were blocked using 5% BSA. Membranes were blotted with the following primary antibodies: anti-JNK1, 2, 3 antibody (Abcam: ab225572), anti-NF-KB p65 antibody (Abcam: ab16502), anti-p38 MAPK antibody (Cell Signaling: 9212s), and anti-beta Actin antibody (Abcam: ab49900). The chemiluminescence was detected using ECL Western blotting substrate (Pierce Biotechnology, Pittsburgh, PA, USA) as described by the manufacturer, and blots were imaged using the ChemiDoc MP Imaging System (Bio-Rad, Hercules, CA, USA). The resulting bands were quantified using ImageJ software. Bands’ intensities normalized to β-actin were used to determine the relative protein expression.

### 4.8. Statistical Analysis

The data were analyzed using GraphPad Prism software 8. One-way ANOVA followed by Tukey’s post-hoc comparison test was used to compare the differences between the groups, and difference with *p* value < 0.05 was considered significant.

## 5. Conclusions

Gold nanorods are promising nanomedical candidates. In this report, GNR decorated with a phospholipid or cholesterol moiety revealed anti-angiogenesis activity and toxicity at the early stages of the normal development of the embryo. The expression patterns of several regulatory factors involved in angiogenesis, apoptosis, and cellular energy metabolism were significantly modulated in our experimental models. Furthermore, both GNR treatments deregulated the JNK1/2/3, NF/KB, and P38 MAPK signaling pathways. Thus, we herein provide evidence that surface-modified GNR could prevent cancer progression and exert promising anti-cancer activity while inducing toxicity at the early stage of embryonic development.

## Figures and Tables

**Table 1:** The mortality rate of the chicken embryos upon treatment with GNR (A) and (B) compared to the control.

Group	Sample Size	Mortality Rate (%) on Day 4 of Exposure
GNR (A)	56	61.4
GNR (B)	42	33.5
Control	25	16.0

**Table 2:** Primer sets used for RT-PCR Amplification.

No.	Gene	Forward Primer (5′-3′)	Reverse Primer (5′-3′)
1	*ATF-3*	AAAAGCGAAGAAGGGAAAGG	ATACAGGTGGGCCTGTGAAG
2	*VEGFC*	AGGGAACACTCCAGCTCTGA	CTCCAAACTCTTTCCCCACA
3	*FOXA-2*	GACCTCTTCCCCTTCTACCG	AGGTAGCAGCCGTTCTCAAA
4	*RIPK1*	CCGTACAGAATTGCAGCAGA	TTCCATTAGCACACGAGCTG
5	*TNFRSF21*	GTGGGCTGATGGAAGACAC	CAGGAGAGCGGAATTCTCAA
6	*TP53BP2*	GTTGTGTTGAGGTGGGTGTC	CATCACGTCCAACCATCGAC
7	*GSS*	AGGGATAGCGACAGATGGTG	TGTTTCTGTGGAGCCTCGAT
8	*HK1*	CATACAGAGCAGCGGAACAC	GTCACTTCTGATGGCAGCAA
9	*GAPDH*	CCTCTCTGGCAAAGTCCAAG	CATCTGCCCATTTGATGTTG

## Data Availability

The data presented in this study is contained within the article.
